# Efficacy of Electroacupuncture Compared to Standard and Manual Needling Therapy for Nonspecific Low Back Pain: A Systematic Review and Meta-Analysis

**DOI:** 10.7759/cureus.72577

**Published:** 2024-10-28

**Authors:** Daniel Hsieh, Yueh-Chi Chen, Hui-Chin Chang, Cheng-Chung Wei, Tsung-Hsien Lee

**Affiliations:** 1 Department of Medical Education, Chung Shan Medical University Hospital, Taichung, TWN; 2 Department of Physical Therapy, Chung Shan Medical University, Taichung, TWN; 3 Department of Physical Therapy, Chung Shan Medical University Hospital, Taichung, TWN; 4 Evidence-based Medicine Center, Chung Shan Medical University Hospital, Taichung, TWN; 5 Department of Library, Chung Shan Medical University Hospital, Taichung, TWN; 6 Institute of Medicine, Chung Shan Medical University, Taichung, TWN; 7 Department of Obstetrics and Gynecology, Chung Shan Medical University Hospital, Taichung, TWN

**Keywords:** acupuncture, electroacupuncture, low back pain, nonspecific low back pain, pain, pain management

## Abstract

Nonspecific low back pain is prevalent, and electroacupuncture is one potential treatment option. However, a focused evaluation of previous experimental studies is lacking. This systematic review and meta-analysis aimed to assess the efficacy of electroacupuncture for nonspecific low back pain in comparison to standard therapy and manual needling therapy. We systematically searched PubMed, Cochrane Library, and Embase up to December 1, 2023, for randomized controlled trials on electroacupuncture for nonspecific low back pain. Outcomes measured included the Visual Analog Scale and Numerical Rating Scale. The risk of bias was assessed using the Cochrane Risk of Bias 2.0 tool. 10 reports were included in the meta-analysis. Six studies used electroacpuncture with standard therapy compared to standard therapy alone. Four studies compared electroacupuncture with manual needling therapies. Compared to control groups, electroacupuncture with standard therapy resulted in a statistically significant large effect in reduction in pain (standardized mean difference (SMD) -0.83, 95% CI -1.22; -0.43, P < 0.0001) Electroacupuncture did not produce a statistically significant result compared to manual needling therapies (SMD 0, 95% CI -0.71; 0.71, P = 1.0) In terms of long-term analgesic effects, electroacupuncture with standard therapy versus standard therapy yielded a statistically significant large effect in reduction in pain at one month (SMD -0.84, 95% CI -1.30 to -0.37, P = 0.0004) and small effect at two months (SMD -0.40, 95% CI -0.65 to -0.16, P = 0.001) after treatment completion. Limitations, including small numbers of included studies and participants, the difficulty of blinding, and clinical heterogeneity among studies, were found. The certainty of evidence for these comparisons remained very low, which highlights the need for more robust studies. Compared to standard therapy alone, electroacupuncture used with standard therapy produced greater pain reduction for nonspecific low back pain in immediate effects and one month and two months after treatment completion. The findings suggest that electroacpuncture may provide sustained pain relief for nonspecific low back pain when combined with standard therapy.

## Introduction and background

Low back pain has been one of the leading causes of health loss for nearly three decades [1]. The prevalence of low back pain in the general population of adults has shown its prevalence at the beginning to be approximately 12%, with a prevalence of 23% at one month, a prevalence of 38% at one year, and 40% to 80% of adults experience at least one episode of back pain during a lifetime [2,3]. Risk factors associated with low back pain include older age, female sex, higher BMI, lower socioeconomic status, smoking, occupational factors, mental stress, depression, sleep deprivation, tiredness, and pain in other parts of the body [1,3-7].

Low back pain leads to a high socioeconomic burden. The hospitalization rate, direct costs, and total costs per patient were 3.2%, USD 9,231 and USD 10,143.1, respectively, in high-income countries [8]. In low- and middle-income countries, the hospitalization rate ranges between 13.4% and 18.7% of patients with low back pain and costs USD 1,226.25 per patient annually [9]. The nature of low back pain could also result in less quantifiable costs such as difficulties doing domestic chores, caregiving, engaging in recreational activities, struggles with relationships, depression, and anxiety [10].

By 2050, the total number of cases of low back pain is expected to increase by 36% to 843 million people, the highest expected increase occurring on the continents of Africa and Asia, largely due to population growth and ageing [11,12].

The current standard treatment for low back pain comprises patient education, non-pharmacologic treatments (e.g., exercise, psychological intervention, and physical therapy), and pharmacologic treatments [13-17]. The American College of Physicians (ACP) and the Canadian guideline suggest starting with non-pharmacologic treatments, including exercise, multidisciplinary rehabilitation, and acupuncture, and consider pharmacologic treatments if first-line treatment produces an inadequate response [14,16].

Acupuncture has been recommended as a non-pharmacologic treatment for low back pain by the North American Spine Society, ACP, and WHO [11,16,18]. Electroacupuncture has been established as a treatment option for low back pain in China [19].

Lam et al. evaluated the efficacy of manual acupuncture and electroacupuncture for nonspecific chronic low back pain, in which electroacupuncture demonstrated a greater reduction in pain intensity compared to usual care [20]. The study compared electroacupuncture alone to usual care alone, rather than comparing electroacupuncture combined with usual care to usual care alone. Asano et al. evaluated the efficacy of acupuncture (manual acupuncture and electroacupuncture) relative to standard therapy for the treatment of nonspecific chronic low back pain [21]. However, electroacupuncture was not analyzed individually from manual acupuncture. Therefore, the efficacy of electroacupuncture could not be demonstrated. Wu et al. evaluated the efficacy and safety of different acupuncture therapies for acute low back pain, and a network meta-analysis showed that electroacupuncture was better than manual acupuncture in reducing Visual Analog Scale (VAS) scores [22]. The study included only those studies that reported VAS scores, and the efficacy of electroacupuncture compared to standard therapy was not demonstrated in the study.

Mu et al. reviewed 37 articles that evaluated the therapeutic effects of acupuncture on nonspecific chronic low back pain, five of which investigated the comparative effects of acupuncture versus electroacupuncture. The findings were inconclusive in terms of the effects on the reduction of pain intensity [23]. Baroncini et al. reviewed 44 studies in a network meta-analysis evaluating the therapeutic effects of acupuncture on chronic aspecific low back pain. Electroacupuncture was not incorporated into the VAS score plots. Therefore, the comparative effects of electroacupuncture on pain intensity were not demonstrated in this review [24].

To our knowledge, this is the first systematic review with meta-analysis to compare the efficacy of electroacupuncture against standard therapy and manual needling therapies for the treatment of nonspecific low back pain. The primary objective of this review is to evaluate current evidence, in the last 20 years, for the efficacy of electroacupuncture in nonspecific low back pain.

## Review

Methods

This review followed the guidelines outlined in the Preferred Reporting Items for Systematic Reviews and Meta-Analyses (PRISMA) Statement [25] and the Cochrane Handbook for Systematic Reviews and Interventions [26]. Our protocol was retrospectively registered in PROSPERO (CRD42024541800).

Eligibility Criteria

Design: Only randomized controlled trials were eligible for inclusion, while studies lacking a comparison group, retrospective studies, and those not involving an intervention (e.g., commentaries and letters to the editor) were excluded.

Population: Eligible studies included participants of either sex with nonspecific low back pain [27]. Age, duration of low back pain, results of the straight leg raise test, and status of previous invasive intervention of the participants did not affect the eligibility of the studies. Participants with specific spinal pathology (fractures, tumors, inflammatory, rheumatologic, or infectious diseases of the spine) who were pregnant or undergoing concurrent acupuncture were excluded.

Intervention and comparison: Eligible studies evaluated the efficacy of electroacupuncture in patients with nonspecific low back pain. Electroacupuncture was defined as the procedure of inserting fine needles into specific acupuncture points (acupoints) and then applying micropulse currents for desired sensations [28].

Eligible studies compared electroacupuncture with standard therapy or manual needling therapies. Standard therapy consisted of non-pharmacological treatments, including exercise, physical therapy, and education on low back pain. Manual needling therapies included acupuncture techniques that did not employ electric currents.

Outcomes: Pain intensity was assessed as a primary or secondary outcome using a standardized quantitative tool. For pain intensity, Chapman et al. and Haefeli et al. recommended the VAS and Numerical Rating Scale (NRS) for their ease of administration and responsiveness [29,30]. VAS and NRS were self-reported results. The rescaling of VAS and NRS for meta-analysis of treatment for low back pain was supported by a review by Wewege et al. [31].

Studies that solely measured descriptive pain intensities, subjective pain opinions, or assessed other pain-related outcomes (e.g., knowledge about pain and quality of life) were excluded. In summary, only studies that measured the outcome with VAS or NRS were included.

Data Sources and Searches

We systematically searched PubMed, the Cochrane Library, and Embase for randomized controlled trials from the date of their inception to 1 December 2023. The search was limited to studies published in English.

Study Selection and Data Extraction

The titles, abstracts, and full texts were independently reviewed by two reviewers (DH and Y-CC). Disagreements were settled through discussions with the third author (THL). Studies were included if they met the following criteria: (I) types of studies: RCTs; (II) participants: patients were diagnosed with nonspecific low back pain; (III) interventions: electroacupuncture; (IV) results: studies must include at least one of the following outcomes: VAS score or NRS score.

The following were excluded: (I) duplicated literature; (II) protocol, case reports, reviews, meta-analyses, conference abstracts, and animal experiments; (III) studies without sufficient data.

DH extracted data with the Cochrane Data Extraction Checklist and YC Chen evaluated the precision of the data. Discrepancies were resolved by reviewing and discussing the article with the third author (THL).

Quality Assessment

The risk of bias in the included studies was assessed using the Cochrane Risk of Bias 2.0 tool template for randomized trials [32]. Methodological quality was evaluated by two independent reviewers (DH and Y-CC). The five domains of this template are as follows: randomization, deviations from intended interventions, missing outcome data, outcome measurement, and selection of reported results. We categorized the overall risk of bias as low, some concerns, or high. If a study received a high-risk rating in any single domain, it was classified as having a high overall risk of bias. Any conflicts between reviewers were resolved through discussion.

Measurement of Intervention Effect

Pain intensity was recorded with two self-reported measurements, a NRS and a VAS. For continuous results, standardized mean difference (SMD) with a 95% CI was used. The use of SMD was appropriate because the included studies assessed pain using different scales, requiring standardization for comparison [33]. An SMD of 0.2 indicated a small effect, 0.5 a moderate effect, and 0.8 a large effect [34]. For studies that reported incomplete pain outcomes (e.g., means without SD), we calculated the necessary results accordingly.

Heterogeneity among the studies was assessed using a χ² test and the I² statistic, as outlined in the Cochrane Handbook [32]. We interpreted the I² values according to the Cochrane Handbook: 0% to 40% indicates likely no important heterogeneity, 30% to 60% signifies moderate heterogeneity, 50% to 90% suggests substantial heterogeneity, and 75% to 100% reflects considerable heterogeneity [32].

Data Synthesis

The meta-analyses were conducted using Review Manager 5.4.1 (The Cochrane Collaboration, 2020). A random-effects model was employed for all analyses due to variations in methodology and scales among the included studies as well as the levels of heterogeneity observed in most of our analyses. For each analysis, we created forest plot graphs, with the area to the left of the center (<0) representing a favorable outcome for the intervention.

We examine the effect of the intervention by the time of outcome measurement, including treatment endpoints, postintervention follow-ups, and comparison categories. We assessed publication bias using Doi plots and the LFK index [35]. The generation of the results was performed using Review Manager 5.4.1 software, JASP software 0.19.0 (JASP Team, 2024), and MetaXL version 5.3 (Epigear International, 2019).

Certainty of Evidence

We evaluated the certainty of the body of evidence in this meta-analysis according to the Grading of Recommendations Assessment, Development, and Evaluation (GRADE) approach [36]. We used the 5 GRADE considerations (risk of bias, consistency of effect, imprecision, indirectness, and publication bias) to assess the certainty of the body of evidence for each result and draw conclusions about the certainty of evidence within the text of the review [36].

Results

**Figure 1:**
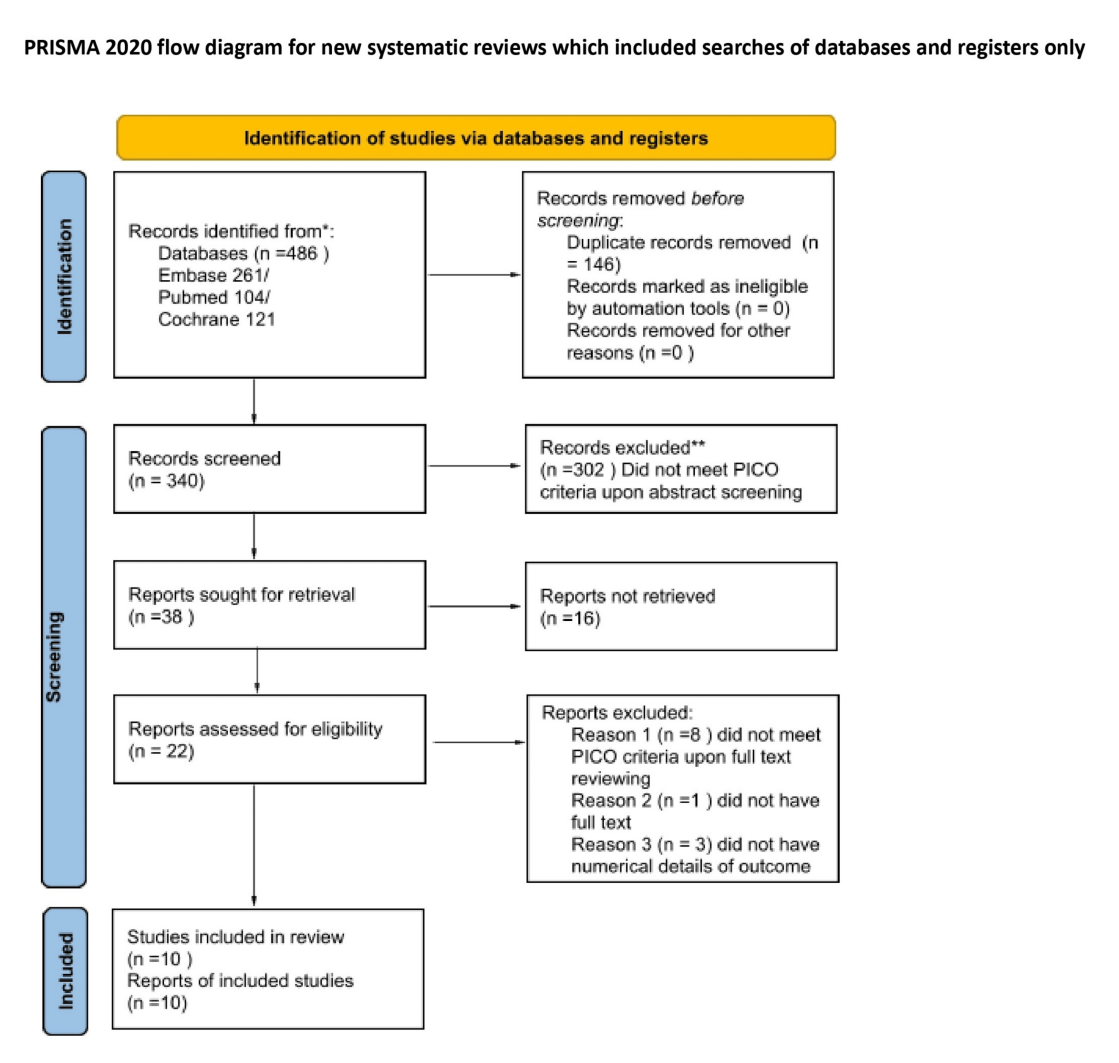
PRISMA flow diagram of the study selection PRISMA, Preferred Reporting Items for Systematic Reviews and Meta-Analyses

Out of 486 records identified from the databases, 146 duplicates were removed. We screened 340 records based on title and abstract, eliminating 302. Of the 38 reports sought for retrieval, 16 could not be obtained. We reviewed the remaining 22 full-text articles and excluded nine, resulting in 13 articles that met the inclusion criteria. However, three of these were excluded from our meta-analysis due to design issues or incomplete or missing pain data [37-39]. In summary, 10 reports were included in our meta-analysis [40-49]. Figure 1 provides the PRISMA flow diagram of the data selection process.

Characteristics of Included Studies

Participants: The 10 included studies were all randomized controlled trials and had 660 participants. The sexual distribution in the intervention group and the control group did not have statistically significant differences (P = 0.09) Studies were conducted in four areas, including Brazil [40,43-45], China [48,49], Hong Kong [46,47], and South Korea [41,42]. Table 1 provides a descriptive summary of the included studies.

**Table 1:** Descriptive summary of the studies included EA, electroacupuncture; MNT, manual needling therapies; NRS, Numerical Rating Scale; RCT, randomized controlled trial; UC, usual care; VAS, Visual Analogue Scale

Study	Location	Design	Sample size (male/female)		Intervention	EA variables	Comparison	Outcome	Outcome measurement
			T	C		Electric current frequency/time; Total sessions; Treatment x frequency; Acupoints chosen			
Comachio et al. (2020) [40]	Brazil	RCT	33 (14/19)	33 (10/23)	EA	10 Hz/10 min 12 sessions 2x/week x 6 weeks BL23, BL30, GV4	MNT	NRS	Baseline
									Six weeks (postintervention)
									Three-month follow-up
Heo et al. (2018) [41]	South Korea	RCT	18 (9/9)	21 (10/11)	EA + UC	50 Hz/15 min 8 sessions 2x/week x 4 weeks EX-B2 (L3-L5) + 0~9 sites selected by personnel	UC	VAS	Baseline
									Four weeks (postintervention)
									Four-week follow-up
									Eight-week follow-up
Heo et al. (2021) [42]	South Korea	RCT	54 (27/27)	54 (26/28)	EA + UC	50 Hz/15 min 8 sessions 2x/week x 4 weeks EX-B2 (L3-L5) + 0~9 sites selected by personnel	UC	VAS	Baseline
									Four weeks (postintervention)
									Four-week follow-up
									Eight-week follow-up
Leite et al. (2018) [43]	Brazil	RCT	17 (7/10)	18 (8/10)	EA	10 and 100 Hz/30 min 10 sessions 3x/week x 3 weeks + 1 day BL22, BL26, BL50, BL53	MNT	NRS	Baseline
									Three weeks (postintervention)
Depaoli et al. (2021) [44]	Brazil	RCT	16 (6/10)	16 (5/11)	EA + exercise	10 Hz/ 20 min 12 sessions 3x/week x 4 weeks BL22, BL26	Exercise	VAS	Baseline
									Four weeks (postintervention)
									One-month follow-up
Torres et al. (2023) [46]	Brazil	RCT	75 (17/58)	25 (15/10)	EA	25, 50, 75 Hz/30 min 10 sessions 2x/week x 5 weeks BL23, BL25, BL40, SP6, KI3	MNT	NRS	Baseline
									Five weeks (postintervention)
Tsui and Cheing (2004) [47]	Hong Kong	RCT	14 (3/11)	14 (5/9)	EA + exercise	2 and 15 Hz/20 min 8 sessions 2x/week x 4 weeks BL25, BL26, GB30, ST36	Exercise	NRS	Baseline
									Four weeks (postintervention)
									One-month follow-up
Yeung et al. (2003) [48]	Hong Kong	RCT	26 (4/22)	26 (5/21)	EA + exercise	2 Hz/30 min 12 sessions 3x/week x 4 weeks BL23, BL25, BL40, SP6	Exercise	NRS	Baseline
									Four weeks(postintervention)
									One-month follow-up
									Three-month follow-up
Cheng et al. (2023) [49]	China	RCT	30 (14/16)	30 (16/14)	EA + exercise	2 Hz/15-20min 10 sessions 5x/week x 2 weeks BL23, BL25, BL26, BL54	Exercise	VAS	Baseline
									Two weeks (postintervention)
Zhang et al. (2019) [45]	China	RCT	70 (35/35)	7 0(38/32)	EA	No frequency/no time 18 sessions 6x/week x 3 weeks BL23, BL25, EX-B8, GV3	MNT	VAS	Baseline
									Three weeks (postintervention)

Interventions and comparisons: Two studies used electroacupuncture with usual care as an intervention, with the same usual care as comparison [41,42]. Usual care was defined as physical therapy and education on low back pain. Four studies used electroacupuncture with exercise as an intervention compared to exercise alone [44,46-48]. Four studies used electroacupuncture alone as an intervention and manual needling therapies as a comparison [40,43,45,49].

Electroacupuncture protocols varied between studies with respect to insertion sites, frequency and time of electric currents, and duration of treatment. The insertion sites were mainly derived from classical meridian points or determined by the symptoms of the participants and the operating personnel. The frequency of the electric currents ranged from 2 to 100 Hz, and the time of the electric currents ranged from 10 to 30 minutes. The duration of treatment ranged from two to six weeks.

Outcomes: The timing of the outcome measurement varied. All studies included results at baseline and at the end of treatment: one study at two weeks [48], two studies at three weeks [43,49], five studies at four weeks [41,42,44,46,47], one study at five weeks [45], and one study at six weeks [40].

Five studies were followed up at one month [41,42,44,46,47], two studies at two months [41,42], and two studies at three months [40,47] after the intervention. Four weeks, eight weeks, and 12 weeks were considered one month, two months, and three months, respectively.

Quality Assessment: Risk of Bias in Included Studies

**Figure 2:**
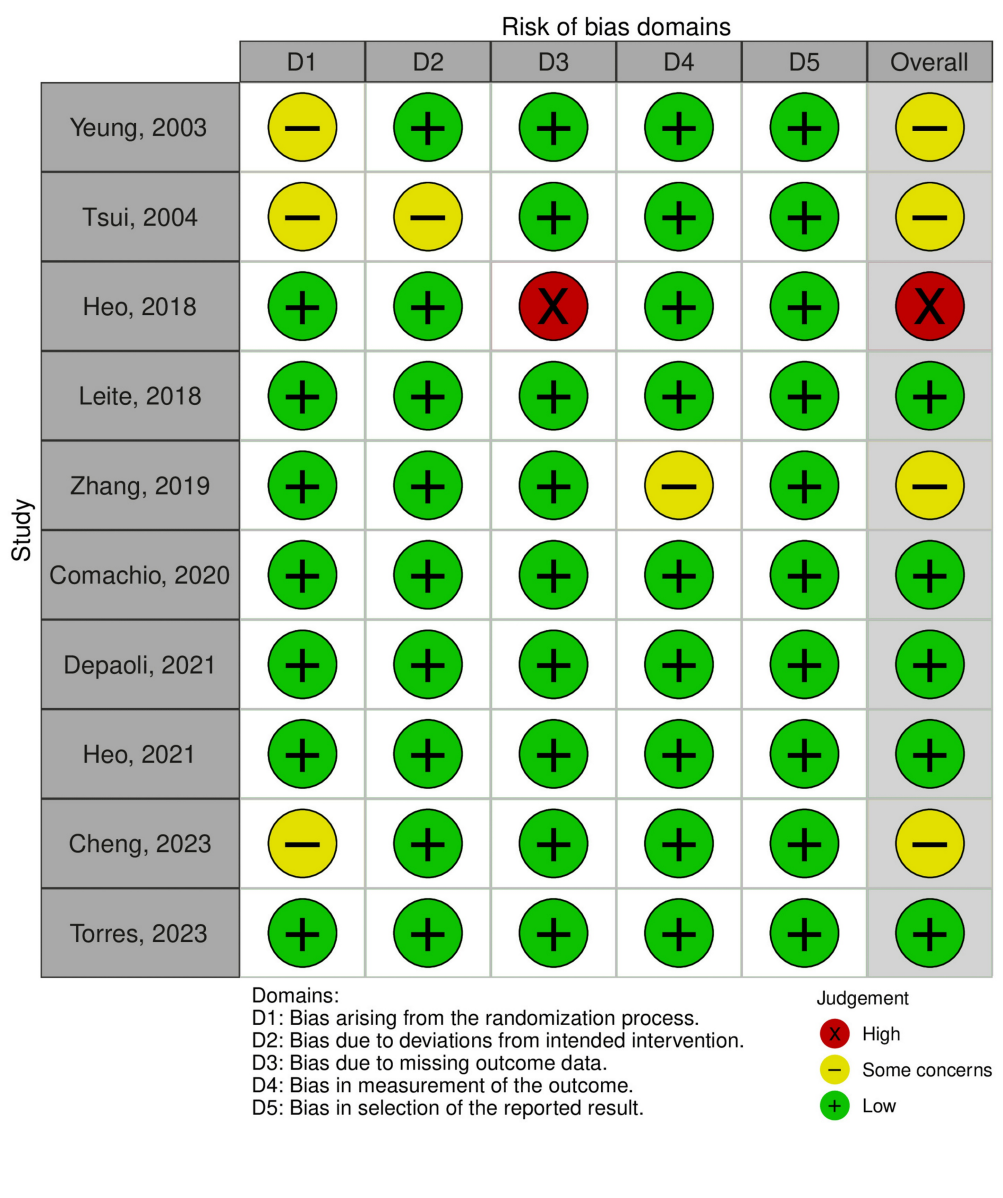
Risk of bias summary D1: bias arising from the randomization process; D2: bias due to deviations from intended intervention; D3: bias due to missing outcome data; D4: bias in measurement of the outcome; D5: bias in selection of the reported result Comachio et al. (2020) [40]; Heo et al. (2018) [41]; Heo et al. (2021) [42]; Leite et al. (2018) [43]; Depaoli et al. (2021) [44]; Zhang et al. (2019) [45]; Torres et al. (2023) [46]; Tsui and Cheing (2004) [47]; Yeung et al. (2003) [48]; Cheng et al. (2023) [49]

Among the 10 studies included in the meta-analysis, five were classified as low risk [40,42-45], four had some concerns [46-49], and one was classified as high risk [41]. The study classified as high risk was due to bias related to the absence of outcome data [41]. The domain most frequently assessed for some concerns was the risk of bias arising from the randomization process, followed by deviations from the intended intervention and measurement of the outcome (Figure 2).

Intervention Effects

The meta-analysis included data from 10 studies with 660 participants. Studies were grouped by timing of outcome measurement or types of comparison.

Standard therapy as a comparison: Six studies (319 participants) used standard therapy, including physical therapy, exercise, and education, as a comparison [41,42,44,46-48]. At the start of the study, there were no statistically significant group differences (P = 0.46). At the end of treatment, a large treatment effect was found (SMD -0.83, 95% CI -1.22 to -0.43, P < 0.01).

Five studies (259 participants) followed up after the intervention [41,42,44,46,47]. These studies measured outcomes 1 month after the completion of treatment [41,42,44,46,47]. In this analysis, a large treatment effect was found (SMD -0.84, 95% CI -1.30 to -0.37, P = 0.0004). Two studies (147 participants) measured outcomes 2 months after treatment completion [41,42]. A small treatment effect was found (SMD -0.39, 95% CI -0.72 to -0.07, P = 0.02).

**Figure 3:**
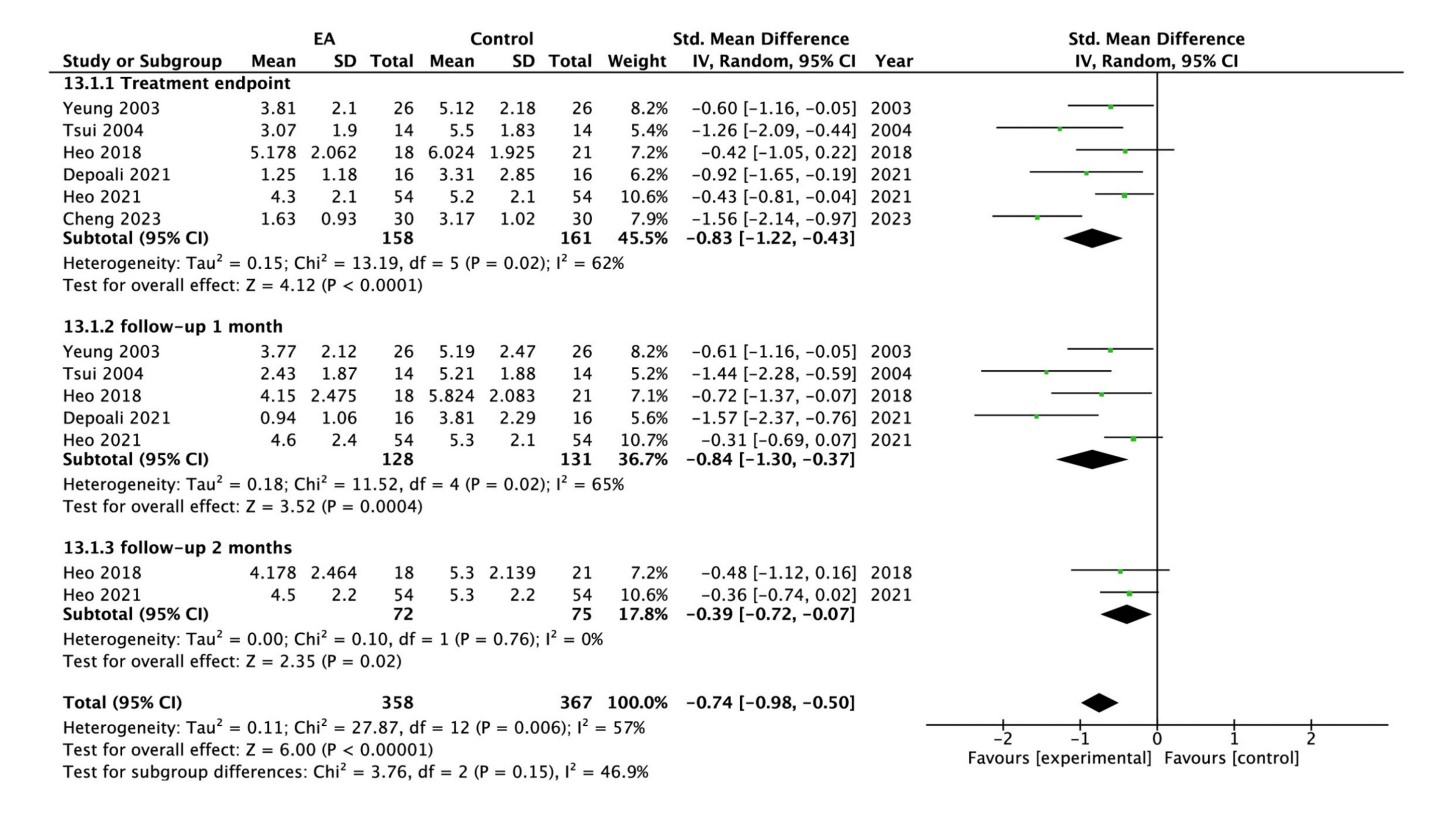
Forest plots of the pain intensities measured at treatment endpoint, follow-up at one month, and follow-up at two months for electroacupuncture with standard therapy compared to standard therapy alone The size of the square represents the weight (%) that the individual subgroup has on the pooled result. SMDs less than zero indicate a treatment benefit. Diamond: summary estimate; solid vertical line: null value EA, electroacupuncture Heo et al. (2018) [41]; Heo et al. (2021) [42]; Depaoli et al. (2021) [44]; Tsui and Cheing (2004) [47]; Yeung et al. (2003) [48]; Cheng et al. (2023) [49]

Figure 3 displays forest plots for outcomes measured at the treatment endpoint, follow-up at one month, and follow-up at two months.

**Figure 4:**
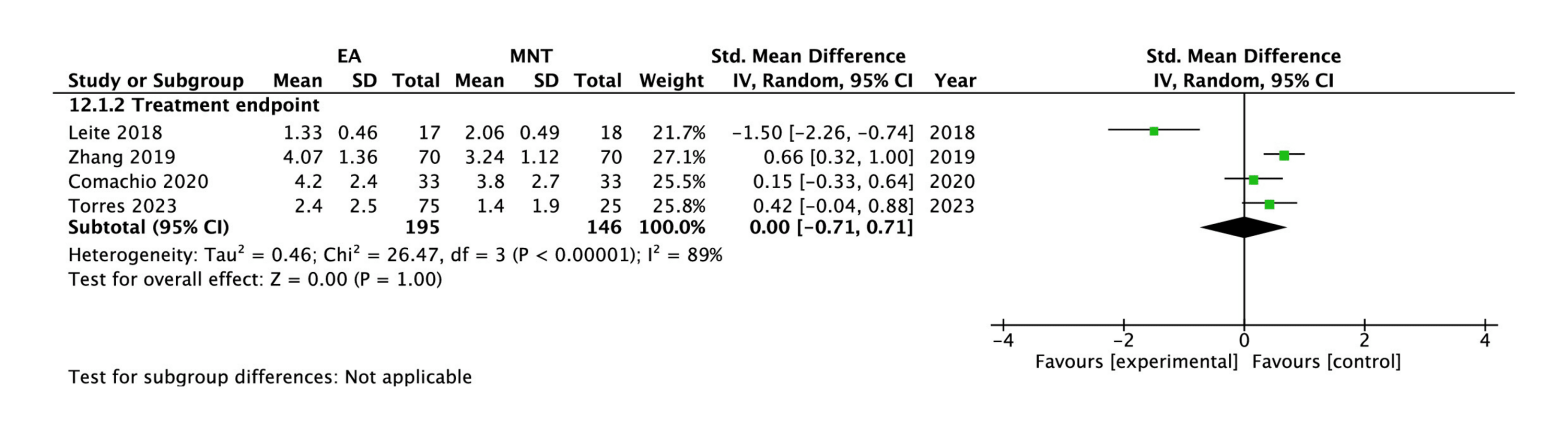
Forest plots of the pain intensities measured at the treatment endpoint for electroacupuncture compared to manual needling therapy The size of the square represents the weight (%) that the individual subgroup has on the pooled result. SMDs less than zero indicate a treatment benefit. Diamond: summary estimate; solid vertical line: null value EA, electroacupuncture; MNT, manual needling therapy Comachio et al. (2020) [40]; Leite et al. (2018) [43]; Zhang et al. (2019) [45]; Torres et al. (2023) [46]

Manual needling therapies as a comparison: Four studies (341 participants) used manual needling therapies as a comparison [40,43,45,49]. At baseline, no statistically significant group differences (P = 0.06) were found. By the endpoint of treatment, insignificant group differences (SMD 0.00, 95% CI -0.71 to 0.71, P = 0.28) with considerable heterogeneity were found (Figure 4).

Publication Bias

**Figure 5:**
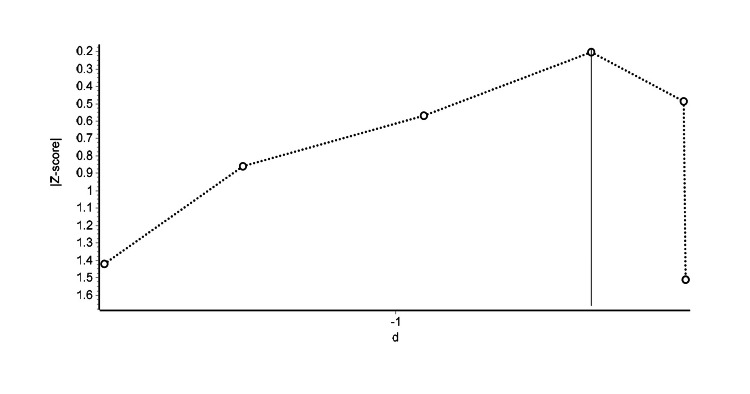
Doi plot of meta-analysis examining the effects of electroacupuncture with standard therapy compared to standard therapy at treatment endpoints Each symbol represents an independent comparison.

Figure 5 presents the Doi plot for the six studies comparing electroacupuncture with standard therapy at treatment endpoints. The LFK index, calculated at -2.15, suggests a potential risk of publication bias, as indicated by the asymmetry in the Doi plot.

**Figure 6:**
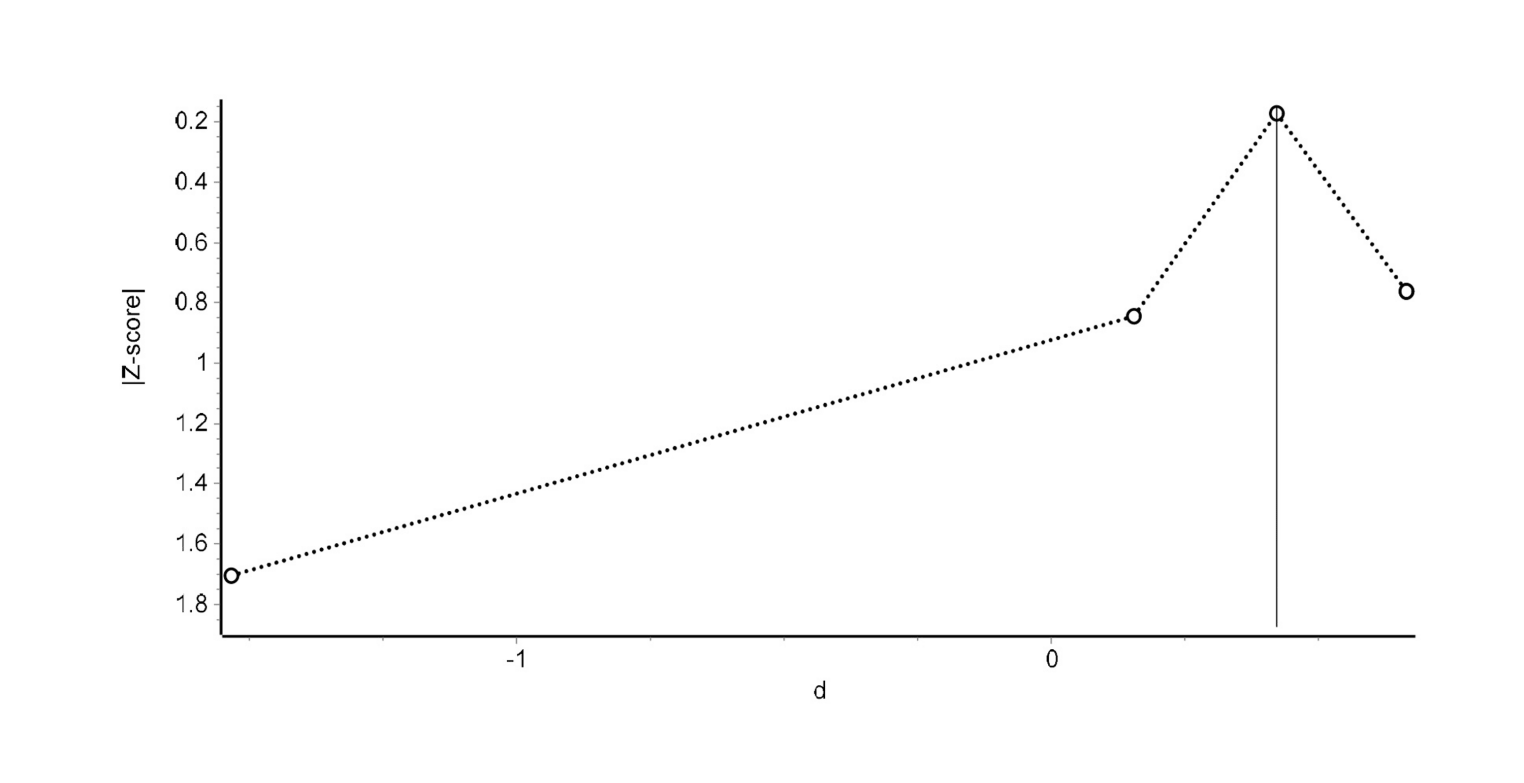
Doi plot of meta-analysis examining the effects of electroacupuncture compared to manual needling therapies at treatment endpoints Each symbol represents an independent comparison.

Figure 6 illustrates the Doi plot for the four studies comparing electroacupuncture to manual needling therapies at treatment endpoints. The LFK index of -2.61 points to a potential publication bias as evidenced by the plot’s asymmetry.

Discussion

This systematic review and meta-analysis included 10 studies investigating the effectiveness of electroacupuncture for nonspecific low back pain compared to standard therapy that included exercise, physical therapy and education, and manual needling therapies.

In this study, two types of comparison were included, which were electroacupuncture with standard therapy compared to standard therapy alone and electroacupuncture compared to manual needling therapies alone. Due to the distinct nature of the comparisons, subgroup analyses were prompted.

Intervention Effect of Electroacupuncture With Standard Therapy Compared to Standard Therapy Alone

Six studies used standard therapy as a comparison [41,42,44,46-48]. Among these studies, five had a treatment duration of four weeks [41,42,44,46,47], while one had a duration of two weeks [48]. The intervention in this group consisted of the standard therapy used in the respective comparison group supplemented with electroacupuncture. A large treatment effect was observed, indicating that electroacupuncture combined with standard therapy resulted in greater pain reduction at the treatment endpoint compared to standard therapy alone.

Five studies measured outcomes one month after finishing treatment [41,42,44,46,47], and a large treatment effect was noted. Two studies assessed outcomes two months after treatment completion [41,42], revealing a small treatment effect. Electroacupuncture with standard therapy provided longer-term benefits at both one month and two months after treatment compared to standard therapy alone. Additionally, one study followed up at three months post-treatment [47], reporting statistically significant differences at that follow-up.

Electroacupuncture combined with standard therapy offered greater benefits than standard therapy alone, both at the end of treatment and during follow-up. This aligns with findings from previous systematic reviews [20]. However, our review incorporates more recent studies. While Lam et al. included studies comparing electroacupuncture to medications, exercise, and sham procedures in a single analysis [20], our review exclusively focused on studies that compared electroacupuncture with standard therapy. This allowed for a clearer demonstration of the efficacy of electroacupuncture when combined with standard therapy.

Intervention Effect of Electroacupuncture Compared to Manual Needling Therapies

Four studies incorporated manual needling therapies as a comparison [40,43,45,49]. In this analysis, no significant group differences were found. Only one study followed up at three months after treatment completion [40], and it did not report statistically significant differences at that follow-up.

The results of the effects of electroacupuncture on pain intensity compared to manual needling therapies in previous systematic reviews were conflicting [22,23]. This review did not find statistically significant group differences. The high heterogeneity can be attributed to the different acupuncture techniques used in the group of manual needling therapies. Future studies with more participants and more consistency in study design may be warranted.

Mechanisms and Targeted Acupoint Analysis in Electroacupuncture Therapy

Electroacupuncture is hypothesized to alleviate pain through induced changes in bioactive chemicals - including opioids, nociceptin/orphanin FQ, serotonin, norepinephrine, glutamate receptors and transporters, cytokines, and signal molecules - in peripheral injury sites, the spinal cord, and supraspinal structures [17].

The analysis of acupuncture points in this review revealed that BL-23 [40,45,47-49] and BL-25 [45-49] were the most frequently utilized acupuncture points, each appearing in five studies. Following these, BL-26 was used in four studies [43,44,46,48]. This finding is consistent with current knowledge of acupuncture points for low back pain [19,28]. A review by Kim et al. found that the BL is the most important meridian for the treatment of low back pain, followed by the GB and GV meridians [28]. Other acupuncture points employed in this review that were frequently selected for the treatment of low back pain, as indicated by Kim et al. [28], include EX-B2 [41,42], GB30 [46], BL40 [45,48], and ST36 [46].

Implications for Future Practice

Electroacupuncture combined with standard therapy, compared to standard therapy alone, demonstrated greater relief from nonspecific low back pain in immediate effects and long-term effects at one month and two months after completion of treatment. The results of this meta-analysis may aid in the future decision-making process when treating patients with nonspecific low back pain.

To our knowledge, this is the first meta-analysis to focus on the efficacy of electroacupuncture compared to standard therapy and manual needling therapies for patients with nonspecific low back pain with follow-up after completion of treatment. Furthermore, our meta-analysis included more racially diverse patients, including Latin Americans and East Asians, thus increasing the applicability among different racial populations.

Certainty of Evidence

Table 2 presents the certainty of the body of evidence in this meta-analysis according to the GRADE approach. Each outcome was assessed individually, and conclusions were drawn about the certainty of the evidence.

**Table 2:** GRADE certainty assessment ^a^ Moderate heterogeneity (I² = 62%) ^b ^Small number of participants ^c^ Doi plots asymmetry and LFK index less than -1 ^d^ Moderate heterogeneity (I² =65%) ^e^ Heo et al. (2018) [40] was assessed as high risk of bias ^f^ High heterogeneity (I² = 89%) GRADE, Grading of Recommendations Assessment, Development, and Evaluation; SMD, standardized mean difference

Certainty assessment							Number of patients		Effect		Certainty	Importance
Number of studies	Study design	Risk of bias	Inconsistency	Indirectness	Imprecision	Other considerations	Electroacupuncture	Comparison	Relative (95% CI)	Absolute (95% CI)		
Immediate effects of electroacupuncture with standard therapy compared to standard therapy												
6	Randomized trials	Not serious	Serious^a^	Not serious	Serious^b^	Publication bias strongly suspected^c^	158	161	-	SMD 0.83 SD lower (1.22 lower to 0.43 lower)	⨁◯◯◯ Very low^a,b,c^	Critical
Long-term effects at one month of electroacupuncture with standard therapy compared to standard therapy (follow-up: one month)												
5	Randomized trials	Not serious	Serious^d^	Not serious	Serious^b^	Publication bias strongly suspected^c^	128	131	-	SMD 0.84 SD lower (1.3 lower to 0.37 lower)	⨁◯◯◯ Very low^b,c,d^	Critical
Long-term effects at two months of electroacupuncture with standard therapy compared to standard therapy (follow-up: two months)												
2	Randomized trials	Serious^e^	Not serious	Not serious	Very serious^b^	Publication bias strongly suspected^c^	72	75	-	SMD 0.39 SD lower (0.72 lower to 0.07 lower)	⨁◯◯◯ Very low^b,c,e^	Critical
Immediate effects of electroacupuncture compared to manual needling therapies												
4	Randomized trials	Not serious	Very serious^f^	Not serious	Serious^b^	Publication bias strongly suspected^c^	195	146	-	SMD 0 SD (0.71 lower to 0.71 higher)	⨁◯◯◯ Very low^b,c,f^	Critical

First, although all the included studies were randomized controlled trials, the nature of the intervention made it difficult to fully blind participants. To address this limitation, studies employed blinding during data analysis. Additionally, the outcome measurement was self-reported by patients, minimizing potential bias from the treatment personnel or assessors. Thus, the overall risk of bias remained low despite this limitation. However, one of the two studies with a two-month follow-up was assessed as having a high risk of bias [40], which may have contributed to an elevated risk of bias within this subgroup. Therefore, the subgroup was rated as having a serious risk of bias.

Second, the heterogeneity among studies was high. To account for the heterogeneity, we analyzed the data with random-effects models, and the heterogeneity was explored using subgroup analyses. The heterogeneity among the studies that incorporated electroacupuncture with standard therapy was moderate (I² = 62%). We proposed the heterogeneity derived from the different electroacupuncture protocols between each study, and the duration of low back pain in each study varied. However, electroacupuncture with standard therapy yielded a large treatment effect despite heterogeneity, thus increasing our confidence in the results. The heterogeneity among the studies that used manual needling therapies as the comparison was high (I² = 89%). The small number of studies included was believed to be the reason for the substantial heterogeneity.

Third, it is difficult to assess imprecision when outcomes are reported as SMDs. To achieve an effect size of 0.2, under the usual standards of α = 0.05 and β = 0.20, the total sample size should exceed 400, which is 200 each for the intervention group and the comparison group. However, both subgroups that used standard therapy or manual needling therapies as a comparison had a total of 319 and 341 participants, respectively. Therefore, the risk of imprecision can reduce the quality of the evidence.

In this study, no indirectness was found. Studies directly compared the effects of electroacupuncture with the comparisons. The outcome measurements directly reflect the intensity of the pain.

Finally, the Doi plots and LFK index indicated potential publication bias. Although the number of studies in both subgroups was small, Furuya-Kanamori et al. suggested that Doi plots and the LFK index are more effective than Egger’s regression in detecting publication bias in such cases [35]. The Doi plots for both subgroups showed asymmetry, further supported by LFK index values below -1, indicating that publication bias was highly likely.

This study had several limitations that could lead to the asymmetry in the Doi plots. To begin with, the review focused exclusively on studies published in English. Second, some of the RCTs included had small sample sizes. Third, although most studies were considered to have a low risk of bias, certain studies did not completely blind participants and personnel due to the nature of the treatments. This incomplete blinding may have resulted in exaggerated positive effects, which are more likely to be published, while studies with null or negative results may have been less likely to be published, indirectly contributing to publication bias. Fourth, there was clinical heterogeneity in various study characteristics, including duration of symptoms, duration of treatment, number of treatments, needle manipulation, variable electrical currents administered, and selection of points of acupuncture. This variability can lead to inconsistent or inconclusive findings, which are often less likely to be published, further contributing to the selective reporting of positive outcomes and thus potentially influencing publication bias.

In summary, the certainty of evidence for outcomes, including immediate effects, one-month follow-up, and two-month follow-up of electroacupuncture combined with standard therapy compared to standard therapy alone, was assessed as very low. Similarly, the certainty of evidence for the immediate effects of electroacupuncture compared to manual needling therapies was also very low.

## Conclusions

In this analysis, electroacupuncture stood out as a promising option, offering greater pain relief than standard therapy when the two were combined. The immediate pain relief and sustained effects lasting up to two months gave it an edge over standard therapy alone, suggesting that electroacupuncture could be a valuable addition for treating nonspecific low back pain. However, when electroacupuncture was compared directly with manual needling therapies, the results told a different story. There was no significant difference between the two in terms of immediate pain relief, suggesting that both methods may be similarly effective. Yet, despite this lack of clear superiority, the certainty of evidence for these comparisons remained very low, which highlights the need for more robust studies.

In light of this, future research should not only focus on developing optimal electroacupuncture protocols but also dive deeper into understanding how it compares with manual needling over the long term. With more data, we can better determine whether one approach truly holds a meaningful advantage over the other.

## Appendices

**Table 3:** Search strategy

Database	#	Search syntax	Citations found
Embase	1	('low back pain' OR 'low back pain'/exp OR 'low back pain'/syn OR low back pain)	92,827
	2	('electroacupuncture' OR 'electroacupuncture'/exp OR 'electroacupuncture'/syn OR electroacupuncture)	11,343
	3	('low back pain' OR 'low back pain'/exp OR 'low back pain'/syn OR low back pain) AND ('electroacupuncture' OR 'electroacupuncture'/exp OR 'electroacupuncture'/syn OR electroacupuncture)	263
2) MEDLINE	1	("low back pain") OR ("low back pain"[MeSH]) OR (low back pain)	48,941
	2	("electroacupuncture") OR ("electroacupuncture"[MeSH]) OR (electroacupuncture)	7,597
	3	(("low back pain") OR ("low back pain"[MeSH]) OR (low back pain)) AND (("electroacupuncture") OR ("electroacupuncture"[MeSH]) OR (electroacupuncture))	104
3) Cochrane CENTRAL	1	#1: (low back pain):ti,ab,kw	15,012
	2	#2: MeSH descriptor: [Low Back Pain] explode all trees	6,116
	3	#3: #1 OR #2	15,113
	4	#4: (electroacupuncture):ti,ab,kw	3,657
	5	#5: MeSH descriptor: [Electroacupuncture] explode all trees	1,162
	6	#6: #4 OR #5	3,657
	7	#7: #3 AND #6	121

## References

[1] (2023). Global, regional, and national burden of low back pain, 1990-2020, its attributable risk factors, and projections to 2050: a systematic analysis of the Global Burden of Disease Study 2021. Lancet Rheumatol.

[2] Manchikanti L, Singh V, Falco FJ, Benyamin RM, Hirsch JA (2014). Epidemiology of low back pain in adults. Neuromodulation.

[3] Rubin DI (2007). Epidemiology and risk factors for spine pain. Neurol Clin.

[4] Jia N, Zhang M, Zhang H (2022). Prevalence and risk factors analysis for low back pain among occupational groups in key industries of China. BMC Public Health.

[5] Mutubuki EN, Luitjens MA, Maas ET, Huygen FJ, Ostelo RW, van Tulder MW, van Dongen JM (2020). Predictive factors of high societal costs among chronic low back pain patients. Eur J Pain.

[6] Parreira P, Maher CG, Steffens D, Hancock MJ, Ferreira ML (2018). Risk factors for low back pain and sciatica: an umbrella review. Spine J.

[7] Shiri R, Falah-Hassani K, Heliövaara M (2019). Risk factors for low back pain: a population-based longitudinal study. Arthritis Care Res (Hoboken).

[8] Fatoye F, Gebrye T, Ryan CG, Useh U, Mbada C (2023). Global and regional estimates of clinical and economic burden of low back pain in high-income countries: a systematic review and meta-analysis. Front Public Health.

[9] Fatoye F, Gebrye T, Mbada CE, Useh U (2023). Clinical and economic burden of low back pain in low- and middle-income countries: a systematic review. BMJ Open.

[10] Froud R, Patterson S, Eldridge S (2014). A systematic review and meta-synthesis of the impact of low back pain on people's lives. BMC Musculoskelet Disord.

[11] World Health Organization WHO guideline for non-surgical management of chronic primary low back pain in adults in primary and community care settings. WHO guideline for non-surgical management of chronic primary low back pain in adults in primary and community care settings.

[12] Dionne CE, Dunn KM, Croft PR (2006). Does back pain prevalence really decrease with increasing age? A systematic review. Age Ageing.

[13] Australian Commission on Safety and Quality in Health Care. Low back pain clinical care standard. Low Back Pain Clinical Care Standard.

[14] Korownyk CS, Montgomery L, Young J (2022). PEER simplified chronic pain guideline: management of chronic low back, osteoarthritic, and neuropathic pain in primary care. Can Fam Physician.

[15] National Institute for Health and Care Excellence. Low back pain and sciatica in over 16s. Low back pain and sciatica in over 16s.

[16] Qaseem A, Wilt TJ, McLean RM (2017). Noninvasive treatments for acute, subacute, and chronic low back pain: a clinical practice guideline from the American College of Physicians. Ann Intern Med.

[17] Zhang R, Lao L, Ren K, Berman BM (2014). Mechanisms of acupuncture-electroacupuncture on persistent pain. Anesthesiology.

[18] Evidence-based clinical guidelines for multidisciplinary spine care: diagnosis and treatment of low back pain. Society.

[19] Liu BY (2010). Acupuncture treatment for low back pain in China. Jpn Acupunct Moxibustion.

[20] Lam M, Galvin R, Curry P (2013). Effectiveness of acupuncture for nonspecific chronic low back pain: a systematic review and meta-analysis. Spine (Phila Pa 1976).

[21] Asano H, Plonka D, Weeger J (2022). Effectiveness of acupuncture for nonspecific chronic low back pain: a systematic review and meta-analysis. Med Acupunct.

[22] Wu B, Yang L, Fu C, Jian G, Zhuo Y, Yao M, Xiong H (2021). Efficacy and safety of acupuncture in treating acute low back pain: a systematic review and bayesian network meta-analysis. Ann Palliat Med.

[23] Mu J, Furlan AD, Lam WY, Hsu MY, Ning Z, Lao L (2020). Acupuncture for chronic nonspecific low back pain. Cochrane Database Syst Rev.

[24] Baroncini A, Maffulli N, Eschweiler J, Molsberger F, Klimuch A, Migliorini F (2022). Acupuncture in chronic aspecific low back pain: a Bayesian network meta-analysis. J Orthop Surg Res.

[25] Page MJ, McKenzie JE, Bossuyt PM (2021). The PRISMA 2020 statement: an updated guideline for reporting systematic reviews. BMJ.

[26] Higgins Higgins, J.P.T. and Thomas, J. J., Chandler Chandler, J. J. Cochrane Handbook for Systematic Reviews of Interventions Version 6.4. https://training.cochrane.org/handbook.

[27] Luo M, Song B, Zhu J (2020). Electroacupuncture: a new approach for improved postoperative sleep quality after general anesthesia. Nat Sci Sleep.

[28] Kim G, Kim D, Moon H (2023). Acupuncture and acupoints for low back pain: systematic review and meta-analysis. Am J Chin Med.

[29] Chapman JR, Norvell DC, Hermsmeyer JT, Bransford RJ, DeVine J, McGirt MJ, Lee MJ (2011). Evaluating common outcomes for measuring treatment success for chronic low back pain. Spine (Phila Pa 1976).

[30] Haefeli M, Elfering A (2006). Pain assessment. Eur Spine J.

[31] Wewege MA, Jones MD, Williams SA, Kamper SJ, McAuley JH (2022). Rescaling pain intensity measures for meta-analyses of analgesic medicines for low back pain appears justified: an empirical examination from randomised trials. BMC Med Res Methodol.

[32] Higgins JP, Savović J, Page MJ, Elbers RG, Sterne JA (2024). Chapter 8: Assessing risk of bias in a randomized trial [last updated October 2019]. Cochrane Handbook for Systematic Reviews of Interventions version 6.5.

[33] Deeks JJ, Higgins JP, Altman DG (2023). Chapter 10: Analysing data and undertaking meta-analyses. Cochrane Handbook for Systematic Reviews of Interventions Version 6.4 (updated August 2023).

[34] Schünemann HJ, Vist GE, Higgins JP (2024). Chapter 15: Interpreting results and drawing conclusions [last updated August 2023]. Cochrane Handbook for Systematic Reviews of Interventions Version 6.5.

[35] Furuya-Kanamori L, Barendregt JJ, Doi SA (2018). A new improved graphical and quantitative method for detecting bias in meta-analysis. Int J Evid Based Healthc.

[36] Schünemann HJ, Higgins JP, Vist GE, Glasziou P, Akl EA, Skoetz N, Guyatt GH (2024). Chapter 14: Completing ‘Summary of findings’ tables and grading the certainty of the evidence [last updated August 2023]. Cochrane Handbook for Systematic Reviews of Interventions Version 6.5.

[37] Lehmann TR, Russell DW, Spratt KF (1983). The impact of patients with nonorganic physical findings on a controlled trial of transcutaneous electrical nerve stimulation and electroacupuncture. Spine (Phila Pa 1976).

[38] Lehmann TR, Russell DW, Spratt KF (1986). Efficacy of electroacupuncture and TENS in the rehabilitation of chronic low back pain patients. Pain.

[39] Meng XY, Bu L, Chen JY, Liu QJ, Sun L, Li XL, Wu FX (2022). Comparative effectiveness of electroacupuncture VS neuromuscular electrical stimulation in the treatment of chronic low back pain in active-duty personals: a single-center, randomized control study. Front Neurol.

[40] Comachio J, Oliveira CC, Silva IF, Magalhães MO, Marques AP (2020). Effectiveness of manual and electrical acupuncture for chronic non-specific low back pain: a randomized controlled trial. J Acupunct Meridian Stud.

[41] Heo I, Hwang MS, Hwang EH (2018). Electroacupuncture as a complement to usual care for patients with non-acute low back pain after back surgery: a pilot randomised controlled trial. BMJ Open.

[42] Heo I, Shin BC, Cho JH (2021). Multicentre randomised controlled clinical trial of electroacupuncture with usual care for patients with non-acute pain after back surgery. Br J Anaesth.

[43] Leite PM, Mendonça AR, Maciel LY (2018). Does electroacupuncture treatment reduce pain and change quantitative sensory testing responses in patients with chronic nonspecific low back pain? A randomized controlled clinical trial. Evid Based Complement Alternat Med.

[44] Depaoli VJ, Selau RC, Blos C (2021). Electroacupuncture and transcutaneous electrical nerve stimulation in chronic nonspecific low back pain: a blind randomized clinical trial. Muscles Ligaments Tendons J.

[45] Zhang J, Wu Y, Li S (2019). Pestle needling at Yāoyángguān-Bāzhèn points for intractable lumbodynia after lumbar disc herniation surgery: a randomized controlled trial. World J Acupunct Moxibustion.

[46] Torres SF, de Macedo AC, Sakai RY (2023). Effect of different frequencies of electroacupuncture on chronic low back pain in older adults: a triple-blind, placebo-controlled, randomized clinical trial. Pain Physician.

[47] Tsui ML, Cheing GL (2004). The effectiveness of electroacupuncture versus electrical heat acupuncture in the management of chronic low-back pain. J Altern Complement Med.

[48] Yeung CK, Leung MC, Chow DH (2003). The use of electro-acupuncture in conjunction with exercise for the treatment of chronic low-back pain. J Altern Complement Med.

[49] Cheng Y, Yu Y, Wang Y, Fan A, Yang H, Wang H, Tang L (2023). Effects of lumbar-pelvic training combined with electroacupuncture on chronic nonspecific low back pain. Medicine (Baltimore).

